# Effects of Divergent Selection for Fear of Humans on Behaviour in Red Junglefowl

**DOI:** 10.1371/journal.pone.0166075

**Published:** 2016-11-16

**Authors:** Beatrix Agnvall, Per Jensen

**Affiliations:** IFM Biology, Avian Behaviour Genomics and Physiology Group, Linköping University, SE 58183 Linköping, Sweden; University of Lethbridge, CANADA

## Abstract

Domestication has caused a range of similar phenotypic changes across taxa, relating to physiology, morphology and behaviour. It has been suggested that this recurring domesticated phenotype may be a result of correlated responses to a central trait, namely increased tameness. We selected Red Junglefowl, the ancestors of domesticated chickens, during five generations for reduced fear of humans. This caused a marked and significant response in tameness, and previous studies have found correlated effects on growth, metabolism, reproduction, and some behaviour not directly selected for. Here, we report the results from a series of behavioural tests carried out on the initial parental generation (P0) and the fifth selected generation (S5), focusing on behaviour not functionally related to tameness, in order to study any correlated effects. Birds were tested for fear of humans, social reinstatement tendency, open field behaviour at two different ages, foraging/exploration, response to a simulated aerial predator attack and tonic immobility. In S5, there were no effects of selection on foraging/exploration or tonic immobility, while in the social reinstatement and open field tests there were significant interactions between selection and sex. In the aerial predator test, there were significant main effects of selection, indicating that fear of humans may represent a general wariness towards predators. In conclusion, we found only small correlated effects on behaviours not related to the tameness trait selected for, in spite of them showing high genetic correlations to fear of humans in a previous study on the same population. This suggests that species-specific behaviour is generally resilient to changes during domestication.

## Introduction

Domestication is one of mankind’s largest biological undertakings, and has served as a proof-of-principle for evolution ever since Darwin [1]. Since the first animals were domesticated about 15000 years ago, dozens of species from different taxa have followed. A striking feature is that a suite of phenotypic changes has tended to evolve independently in several distantly related species, often referred to as the domesticated phenotype [2]. This includes changes in morphology, such as skull shape, bone and pigmentation; physiology, such as reproductive seasonality and stress responses; earlier sexual maturation; and behaviour, such as tameness and increased social tolerance. For example, studies of rats have shown large differences in exploration and aggression [3], neophobia and swimming [4] in domesticated strains compared to wild types. Bengalese finches, a domesticated strain derived from the White-rumped munia (*Lonchura striata*) have a fundamentally altered song pattern [5] and domesticated brown trout (*Salmo trutta*) show changes in their antipredator behaviour [6]. Most strikingly, dogs differ in a range of social and human-oriented behaviour from their wolf ancestors [7].

A central question in this field is whether the common phenotype has developed as a single, correlated complex of traits, perhaps due to similar genetic architectures in different species, or whether it is the result of recurring directed selection by humans of preferred traits. Experimental studies of the domestication process offer one way to distinguish between these explanations [8,9].

Whilst domesticated animals clearly show remarkable differences compared to their ancestors in many different behavioural aspects, it is also obvious that some behaviour appears highly resilient to domestication. For example, studies in semi-natural environments have found that given the opportunity, pigs will show a wide range of the behaviour characteristics of their ancestor, the European wild boar. This includes forming social groups resembling wild boar herds [10] and displaying the full range of functional and ancestral parental behaviour [11]. On the other hand, some parts of domesticated behaviour exhibit large differences from what is seen in the ancestors. For example, feral domesticates often show a highly increased social tolerance with reduced territoriality and larger, more loosely organised social groups [2] and dogs show communicative and cooperative skills against humans which are not found in their ancestors, the wolves [12]. It is not known which aspects of behaviour are dynamic and moldable during the evolutionary rapid domestication process, and which remain more resilient and difficult to change. Since most domestic animals are kept in intensive farm systems for food production, where little attention is paid to the behavioural needs of different species, it is highly important to gain a closer understanding of how natural behaviour has changed during domestication.

In a famous experiment, Belyaev and co-workers selected farmed silver foxes (a melanistic variant of the red fox, *Vulpes vulpes*) for reduced fear of humans, and demonstrated a range of correlated phenotypic responses [13] in colour, skeletal form and reproductive physiology. Similarly, rats [14] and mink [15] have been used for the same type of experiments, and in all three species tameness (the selected trait) increased rapidly. Whereas a number of studies have explored the behavioural differences between wild species and their domesticated variants ([4,16,17]), no assessment has been done specifically of the effects of selection for increased tameness on other, species-specific behaviour not immediately related to fear of humans.

We have focused on the ancestor of domestic chickens, the Red Junglefowl (*Gallus gallus*). This species was domesticated about 8000 years ago [18] and today constitutes the world’s most numerous food producing animal species [19]. Most chickens, regardless of whether they are bred for egg production or meat, are kept under intensive and often highly barren conditions, and their welfare has often been questioned; for example, detrimental behaviour disorders such as feather-pecking and cannibalism are common [20]. Some of these abnormal behaviours have a strong genetic component [21,22] and increased understanding of how behaviour has changed during domestication is therefore of high relevance from an animal welfare perspective.

The population we used for our experiments was derived from zoo animals, and probably carries some unknown degree of introgression from domesticated chickens. However, they are sufficiently different from modern laying hens to serve as a model for the true ancestors [23]. Previously [24], we have found that Red Junglefowl, selected for reduced fear of humans (as a mimic of early domestication conditions) show correlated responses in several unrelated phenotypic traits, in the same way as the earlier mentioned fox, rat and mink experiments. For example, after three to five generations, less fearful Red Junglefowl are larger at hatch, grow faster, have larger offspring, and have an increased basal metabolic rate and feed efficiency [25,26]. This is accompanied by modifications in brain gene expression profiles [27]. Significant genetic correlations between tameness and several functionally unrelated behaviour suggests that correlated selection responses might pertain also to these activities [24]. The present experiment consists of continued studies of the same population. We therefore expect behaviour that is genetically correlated to tameness to have changed over the three generations of selection added to the previous study [24].

Therefore, the aim of the present study was to examine possible correlated effects of selection for reduced fear of humans on unrelated, species-specific behaviour in Red Junglefowl. We focused on fear reactions in general, and on sociality, exploration and foraging.

## Material and Methods

### Ethical note

The experiments were performed according to regulations for animal experiments, and were conducted under license from the 'The Linköping regional committee for ethics in animal research', approval number 122–10. The ethical committee specifically approved this study.

### Animals, breeding and housing

We used Red Junglefowl originating from two different zoo populations, one from Copenhagen and one from Götala research station. They had been kept in our chicken facilities for more than five generations before the start of the experiment. In each generation, 70–80 individuals were maintained per population. More information on the background of the birds can be found in [28].

To obtain the initial outbred parental (P0) generation, we first crossbred birds from the two populations during two generations. More details regarding the breeding scheme can be found in a previous paper [24].

In each generation from P0 onwards, all birds were subjected to a fear-of-human test at 12 weeks of age (described below). Based on the scores in the test, they were divided into a high, low or intermediate group. Birds were selected for breeding within each group, so the most and low fearful from the high and low groups were bred, while birds from the intermediate group were randomly mated (in the following referred to as “unselected”). Details on the selection can be found in an earlier paper [24].

The birds were pedigree hatched and individually wing-tagged, weighed and vaccinated against Marek’s disease. For the first five weeks, they were housed in small floor pens (0.75x0.75 m; from two weeks: 1.5 x 1.5) with wood chips and heaters, and free access to commercial chick feed and water, in groups containing about 30 birds from all selection groups. Here, the room temperature was maintained between 25 and 30°C and light was kept at a level of 10 Lux for 12 h per day. At the age of five weeks, they were moved to the University’s chicken research facility where they were housed in sex separated groups in pens, measuring 3 x 3 x 3 m, containing free access to commercial chicken food and water, nests, perches and wood chips on the floor. In this facility, room temperature was maintained at about 20°C and light levels about 10 Lux during the 12 h daily light period. At all ages, groups were mixed with birds from all three selection lines.

Animals were only handled after hatch for marking, vaccination and weighing, and after that only for weighing at four instances throughout life, for transport from hatchery to adult housing (at five weeks) and for behaviour testing.

### Behavioural tests and recording

Birds from the parental generation (P0; N = 46, of which 23 females and 23 males; only the birds used as parents for the first selected generation were included) and the fifth selected generation (S5; N = 112, of which 46 females and 66 males; all available birds were included) were exposed to a series of behavioural tests, designed to measure important aspects of the normal behaviour of chickens. The tests were carried out at different ages, to encompass the possibility that selection related differences between the lines could either increase or decrease with age and increasing environmental influences. However, the tests were all performed before the onset of sexual maturity, to avoid the problems that might arise when tests collide in time with egg laying. Due to different technical causes, a few birds were not included in some of the tests (0–5 birds per test from each generation). The omitted birds were not the same in all the tests, so in total the N-values given above show the number of birds that were included in at least three of the tests. These tests, as well as a detailed ethogram, have been thoroughly described previously [24] and a summary of the procedures are given below.

All tests were conducted in test arenas indoors, between 10 am and 15 pm. The arenas for the chicks were totally enclosed to avoid all external stimuli, and light was provided in the arenas at a level of about 25 Lux during the tests. For the older birds, tests were carried out in test arenas kept in the same house as the holding pens, with similar light and temperature conditions as in the rest of the building.

#### Fear of humans (FH)

When 12 weeks old birds were placed individually in an arena measuring 100 x 300 x 210 cm. A test person was positioned at the end of the arena. The behaviour of the bird was manually scored using one-zero sampling every 10^th^ second during one minute, while the test person approached the chicken according to a pre-set scheme, ending by attempting to touch it. Based on the behavioural recordings, each bird was given a composite fear score ranging from 20–100, where 20 meant low and 100 high fear of the human. This score was calculated as the average of the individual 10-s scores.

#### Social reinstatement

At three weeks of age, social affiliation (sociality) to conspecifics was assessed in a Social Reinstatement Test. The birds were placed individually in a runway, measuring 20x120x40 cm, with two similar aged unfamiliar stimulus animals from the same selection groups being kept in a compartment at the distant short end, separated from the arena with wire mesh. Birds were introduced to the arena in darkness, and the test commenced with the onset of light. During five minutes we recorded the movements of the chicken with the software EthoVision (Noldus, version 3.1). The test was replicated twice on successive days for the same bird with different stimulus animals. The total time the chicken spent within 20 cm of the stimulus birds (“social zone”) and the total distance it moved during the test was recorded by taking the averages from the replicated tests.

#### Open field (4 weeks)

At the age of four weeks, open field behaviour was assessed as a measure of exploration and general fearfulness. The Open Field-arena measured 80x120x40 cm and was virtually divided into two areas; center (40 x 80 cm), and periphery. The movements in the arena were automatically recorded during five minutes with EthoVision (Noldus, version 3.1), and the test was replicated twice. We recorded total distance moved and the time spent in the periphery, taking the averages from the replicated tests.

#### Foraging and exploration test

At 13 weeks, we assessed behaviour in a Foraging and Exploration test. Birds were individually placed in a 0.9 m^2^ square arena, separated with wire mesh from a surrounding space (2.7m^2^) where four companion birds were kept. The companions did not have any access to food or water during the tests. A cardboard box with three plastic cups fixed into it was placed in each of the four corners of the test arena. One of the cups in each corner contained wood shavings with 20 mealworms hidden therein, while another contained only wood shavings, and the third freely available, familiar chicken food. The birds had been exposed to the same cardboard boxes as used in the test in their home pens, and with being fed mealworms, continuously during one week before the test. Hence they were familiar to the setup and to mealworms at the start of the test.

Following 60 min of food deprivation, one bird at a time was placed in the inner part of the arena with the four companions outside, and the feeding behaviour was recorded through direct observations by a hidden observer during five minutes. We counted the total number of pecks in the cup with mealworms, and number of changes between the corners.

#### Aerial predator

At 15 weeks, a simulated aerial predator attack response was used to assess anti-predator behaviour. Birds were placed one and one in an arena measuring 50 x 150 x 50 cm. The birds were allowed five minutes to habituate, and then a hawk model was slid above the length of the arena starting 160 cm above one end of the arena and descending to 60 cm in the other end in about two seconds. The model consisted of a wooden profile of a hawk, measuring 320 mm length and 640 mm wing-span. See also [29] and [28]. The behaviour was recorded with one-zero sampling using 10 seconds interval and direct recording by a hidden observer for five minutes immediately following exposure. The frequency of Stand Alert (standing vigilant with head raised), Exploration (pecking and scratching at floor and walls) and Freeze (crouching motionless) was recorded.

#### Open Field (16 weeks)

At 16 weeks the animals were subjected to a second open field test, similar to the one at four weeks. This time, the arena measured 190 (w) x 190 (l) x 100 (h) cm with a center zone of 100 x 100 x 100 cm (the rest defined as the periphery zone). By means of direct observations, the time spent in the periphery as well as frequency of crossed zone borders (as a measure of distance moved) were recorded.

#### Tonic Immobility

The tonic immobility reaction [30] was assessed when the birds were 17 weeks. Each bird was gently placed on the back in a wooden cradle, out of sight and hearing of other chickens. A soft pressure was applied to the chest for 10 seconds, and if the chicken remained immobile for at least the 5 seconds following release, it was considered to have entered tonic immobility. If not, the induction attempts were repeated two more times at a maximum. Time until rightening was recorded, with a max time of 600 s allowed, and the number of induction attempts necessary to induce tonic immobility (with 3 attempts as maximum value).

### Statistics

For all variables except fear scores, differences between selection lines were calculated separately within each generation. The data were normalized within generation by dividing the value for each individual with the mean value of the unselected group, and then means and standard errors of these normalized values were calculated for each of the three selection groups in each generation. In this way, the mean for the unselected group was set to 100 for all variables.

The effects of selection group, sex, and the interactions between them were assessed with ANOVA, using General Linear Models. The adequacy of normal distribution was determined by visual inspection and the Shapiro-Wilk test, and when the data deviated significantly from normality the variables were log-transformed (log X+1) before analysis. We only report statistical parameters for the main effects of selection line, and for those other effects which are statistically significant (P<0.05). Data reported are always non-logged. For all the statistical analysis, Statistica version 13 was used.

### Results

There was a significant effect of selection group on the score in the Fear of Human test in both generations (P0, High: 4.0±0.1, Unselected: 3.2±0.1, Low: 2.6±0.1, F_2,40_ = 78.4, P<0.001; S5, High: 3.6±0.1, Unselected: 3.2±0.1, Low: 2.5±0.1,F_2,106_ = 7.38, P<0.001). In the P0-generation, this was obviously an effect of a deliberate splitting of the birds into those with high and low scores, but in S5, this indicates that the continued selection over the five generations had produced lines of birds with significantly different fear scores.

**Fig 1:**
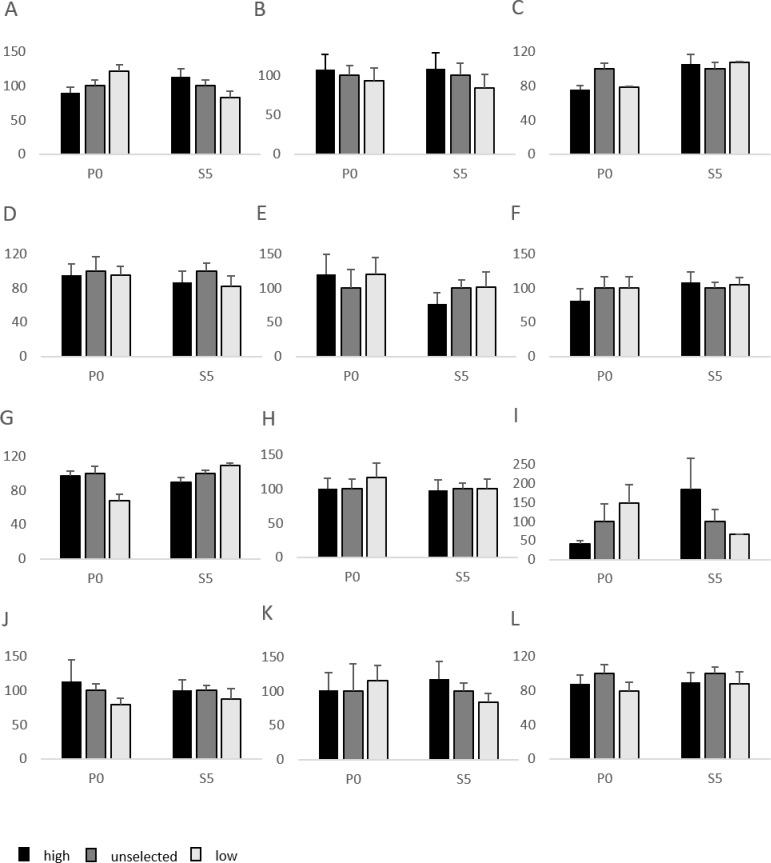
Behaviour responses in the parental (P0) and fifth selected (S5) generation of Red Junglefowl. All data are normalized against the unselected birds in each generation, which are set to 100 for all variables. A. Social reinstatement: total distance moved; B. Social reinstatement: time spent in the social zone; C. Open field at 4 weeks: time spent in periphery; D. Open field at 4 weeks: total distance moved; E. Foraging/exploration: number of pecks to meal worms; F. Foraging/exploration: Changes between food sources; G. Aerial predator: stand alert; H. Aerial predator: exploration; I. Aerial predator: freeze; J. Open field 16 weeks: time spent in periphery; K. Open field 16 weeks: numbers of zone borders crossed; L. Tonic immobility: time to rightening.

In the Social Reinstatement test, there was a significant effect of selection line on total distance moved in the P0 generation, but not in S5 (Fig 1A; P0: F_2,40_ = 3.4, P<0.05; S5: F_2,104_ = 1.7, P = 0.18). Neither in P0 or in S5 were there any significant effects of selection group on time spent in the social zone (Fig 1A and 1B; P0: F_2,40_ = 0.7, P = 0.51; S5: F_2,104_ = 0.4, P = 0.64). However, in S5 there was a significant interaction between sex and selection line for time spent in the social zone (F_2,104_ = 5.2, P<0.01), where males from the low line were less in the zone than females from low line.

In the Open Field (4 weeks) test, there was a significant effect of selection group in P0 on time in periphery (Fig 1C; F_2,40_ = 4.0, P<0.05), but not on total distance moved (Fig 1D; F_2,40_ = 0.04, P = 0.96). In S5, there were no effects on either time in periphery (Fig 1C; F_2,106_ = 0.03, P = 0.97) or total distance moved (Fig 1D; F_2,106_ = 1.5, P = 0.21), while there was again a significant interaction between sex and selection line on both variables (time in periphery: F_2,106_ = 3.3, P<0.05; total distance moved: F_2,106_ = 3.2, P<0.05). Males from the low fear line were more active and spent more time in the periphery than females from the low line.

In the FE-test, there were no significant effects of selection line on any behaviour in either of the generations (Fig 1E and 1F; Number of pecks to meal worms, P0: F_2,38_ = 0.1, P = 0.9; S5: F_2,105_ = 0.25, P = 0.78; Changes between food sources, P0: F_2,38_ = 0.35, P = 0.70; S5: F_2,105_ = 0.43, P = 0.65). There were also no significant interactions between sex and selection line.

In the Aerial Predator test, there were significant effects of selection line on stand alert in both generations (Fig 1G; P0: F_2,40_ = 6.9, P<0.01; S5: F_2,106_ = 3.95, P<0.05). There was a significant interaction between sex and selection line on exploration in P0 (F_2,40_ = 5.8, P<0.01) where males from the low line explored more than females from the low line but there were no effect of selection in S5 (F_2,106_ = 0.05, P = 0.95) (Fig 1H). In P0 there was also significant effects of sex on exploration (F_1,40_ = 5.5, P<0.05), where males explored more. For the behaviour freeze, there were no significant effects in any of the generations (Fig 1I; P0: F_2,40_ = 1.2, P = 0.31; S5: F_2,106_ = 1.29, P = 0.28).

In the Open Field test carried out in the 16 weeks old birds, there were no significant effects of selection line on any of the behaviours recorded (Fig 1J and 1k; time spent in periphery: P0: F_2,40_ = 1.2, P = 0.31; S5: F_2,106_ = 0.70, P<0.50; numbers of zone borders crossed: P0: F_2,40_ = 0.92, P = 0.40; S5: F_2,106_ = 0.31, P = 0.07).

In the tonic immobility test, there were no significant effects of selection on time to rightening in any of the generations (Fig 1L; P0: F_2,40_ = 0.84, P = 0.44; S5: F_2,106_ = 0.28, P = 0.75). However, there was a significant main effect of sex in S5 (F_2,106_ = 5.3, P<0.05) where females took longer to righten. There were no effects of selection on number of attempts to induce tonic immobility in any of the generations (P0: F_2,40_ = 0.65, P = 0.52; S5: F_2,106_ = 0.74, P = 0.48).

## Discussion

In spite of repeated selection for animals showing divergent responses in a fear-of-human test, which produced a clear difference in the trait selected for, the effects on other aspects of functionally unrelated behaviour, such as sociality, foraging and exploration were relatively small. Thus, many aspects of the normal species-specific behaviour appear to have been less affected by increased tameness in the animals studied here compared to effects on other traits demonstrated earlier.

Previously, we have shown that selection for reduced fear of humans in this group of Red Junglefowl is correlated with a number of phenotypic effects resembling those associated with the domesticated phenotype, shared among many different species [2]. In our studies, this includes increased hatch weight, growth and size of offspring, as well as increased metabolic rate and feed efficiency in the low fear birds [25,26]. Furthermore, these differences are associated with modified gene expression patterns in the brains of the selected birds [27]. This would suggest that correlated selection responses may be important in generating the domesticated phenotype, and it has been suggested that tameness may in fact be the driving trait [9,25]. In a previous study, we also found significant heritabilities for several of the behaviour variables recorded in the present experiment and significant genetic correlations with the fear of human score [24]. The reason for why we nevertheless did not find stronger phenotypic correlated effects of selection could possibly be that the present selection was imposed for a too short time (only five generations). Regardless, it is obvious that the behaviour studied here is more resilient to correlated selection responses than other traits, such as growth and reproduction. In previous studies on other species, fundamental behaviour changes have been observed several generations later than what we have studied here (for example, up to 20 generations in farm foxes; [9], and more than 60 generations in rats; [14]). Continued divergent breeding of the selected lines might throw light on the corresponding selection responses in Red Junglefowl.

The results are in line with the well-known fact that fundamental species-specific behaviour is remarkably resilient during domestication, unless specifically targeted for selection. For example, after about 8000 years of domestication, pigs still form strict social groups, forage like ancestral wild boars, build elaborate nests to give birth, and show the full ancestral parental behaviour if given the opportunity [10,17,31]. Present-day domesticated chickens are highly motivated to perch at night-time, stay in social groups, hide their eggs in secluded nests and forage in similar ways as Red Junglefowl [19,32].

Of the tests we applied, neither the Foraging Exploration test nor the Tonic Immobility test showed any effects correlated to selection for tameness. We previously observed no significant heritability or genetic correlation for Tonic Immobility, which is in line with the lack of selection responses. Tonic Immobility is generally considered to be an anti-predator response with moderate heritability [30]. Our results suggest that fear of humans may have a different underlying genetic architecture than the type of fear measured by the Tonic Immobility test. In the Foraging Exploration test, on the other hand, our previous studies found strong heritabilities and genetic correlations with fear of humans, suggesting that we should have observed correlated selection responses [24]. It is possible that the number of generations studied here may be too few to see an effect. It could also be speculated that other factors have over-shadowed any effects on the foraging behaviour; for example, increased fear levels may have affected the general responses in the test situation. We also previously found a correlated response in basal metabolic rate and feed efficiency [25], indicating that some aspects of the feeding behaviour might in fact have been affected, although our present test was not able to show so.

We tested whether sociality would be affected in the five generations of selection studied here by means of the well-established social reinstatement test. This has been used in other previous research, and the responses in this test are highly responsive to genetic selection in quail [33]. Furthermore, in chickens the social reinstatement behaviour has been suggested to be linked to some aspects of fear [34]. However, in our experiment, the correlated responses were weak, and mainly affected males as judged by the significant interaction between selection and sex. In previous studies, we have reported significant heritability and genetic correlation with fear of humans for the activity levels in this test [24]. Although the effects were small in our present experiment, it is possible that more generations of selection would increase the differences between the lines. This is in line with the fact that the numerical figures indicated an opposite relationships in P0 compared with the tendency in S5 (low fear birds moving more than the other lines in P0 and less in S5).

Open field activity is commonly used as a measure of exploration and anxiety (due to the novel environment and social isolation) in chickens as well as in many other species, and has a clear genetic component [30,35]. We tested the birds at two different ages, but in neither case did we observe any effects of selection for tameness. Previously, we found a moderate heritability for open field-behaviour in the present population, but no clear genetic correlations to fear of humans [24].

Finally, we found significant main effects of selection, as well as of sex and their interactions, on behaviour responses to a simulated aerial predator attack. This indicates that the fear of humans that we have selected on might in fact be a general fear of predators trait, so reduced fear of humans would eventually lead to a generally lower alertness towards other perceived predators as well. Domesticated chickens respond differently than Red Junglefowl in a similar test, indicating that domestication may in the long run lead to a modified anti-predator behaviour [29].

In some behaviours we found a significant interaction between sex and selection. This corroborates previous studies of Red Junglefowl, showing that the two sexes differ in their fear behaviour, and that this is probably mediated by different genetic and epigenetic architectures related to fear in males and females {Natt:2014hr}. A possible interpretation of our data is that the sexes show different degrees of correlated selection responses. In this context, it is also interesting to note that among modern, domesticated chickens, the sexes tend to respond differently to stress, which again may be related to the different genetic architectures underlying this response {Natt:2012ch, Ericsson:2016jt}

It is somewhat surprising that the behaviour recorded here did not diverge more according to selection for tameness, in spite of previous observations of high genetic correlations to fear of humans. However, as stated by Gromko [36], pleiotropic effects of genetically correlated loci may vary. Hence, the observed lack of selection effects is probably due to a complex genetic architecture with many loci contributing small effects. As a consequence, these traits would probably only diverge very slowly unless actively selected upon directly. It is also possible that epigenetic differences may have played a role here, since, for example, DNA-methylation can vary immensely between wild and domestic variants and may be important for rapid evolutionary responses [37,38].

It is important to emphasise that the present study had as its starting point an outbred population of zoo-raised Red Junglefowl, and not wild-caught animals. The species is considered endangered and it can even be questioned whether truly wild (not mixed with domesticated chickens) Red Junglefowl exist [18], so it is quite likely that the P0-generation we generated does not represent the true wild form. However, this does of course not fundamentally affect the fact that correlated selection responses in behaviour were sparse. It should also be noted that birds from the different selection lines were housed together, which could possibly have caused them to behave more similarly and thus obscure some selection responses. For example, fearful reactions of birds from the high fear strain could have caused increased fear also in the rest of the birds in the same pens. Therefore, our results possibly underestimate the genetic contribution to differences between selection lines. However, the alternative of housing each selection line separately would have generated other possible confounders (pen effects), which might have caused an over-estimate of the genetic differences, so we decided for the more conservative experimental design.

In conclusion, whilst five generations of selection for reduced fear of humans has caused significant effects on several phenotypic traits associated with the domesticated phenotype, as found previously [25,26], the present results show relatively small effects on other aspects of fearfulness, sociality, exploration and foraging behaviour. This indicates that fundamental species-specific behaviour is more resilient to correlated selection responses in the presently studied Red Junglefowl than phenotypes related to growth and reproduction.

## Supporting Information

S1 Table Data set. The complete data set used for the statistical analysis.
(XLSX)
